# Retrospective Analyses of Stability and Variability in Relative Age Effects of Handball Talents Over Seventeen Years

**DOI:** 10.1186/s40798-024-00797-3

**Published:** 2025-01-23

**Authors:** Jörg Schorer, Dirk Büsch, Irene Faber, Nick Wattie

**Affiliations:** 1 Institute of Sport Science, Carl von Ossietzky University of Oldenburg, Ammerländer Heerstr. 114-118, 26129 Oldenburg, Germany; 2 Faculty of Health Sciences, Ontario Tech University, Oshawa, Canada; 3 Research Centre Human Movement and Education, Windesheim University of Applied Sciences, Zwolle, The Netherlands

**Keywords:** Talent, Expertise Development, Birth-date, Youth

## Abstract

In the last thirty years research on relative age effects (RAEs) has exploded in numbers. However, the stability and variability of these effects have hardly been investigated. The three aims of this retrospective study were first to investigate the stability and variability of RAEs over 17 years, second to compare these effects for young female and male athletes, and third to compare these effects between selected and non-selected athletes relative to variability estimates from 17 years prior to assess possible changes in athlete development trends. For this study, birth dates were provided for all participants of the talent selection camps by the German Handball Federation from 2008 to 2024. Results show that first while some variability was observed, the effects remained stable. Second, there are only small differences between sexes in general, although these increased with selection. And thirdly, that selections create stronger effects for male athletes, but not for female ones. Taken together, this study provides an interesting picture of the variability and stability of relative age effects over 17 years.

## Key Points

• Relative age effects (RAEs) in handball are rather stable over a period of seventeen years.

• There is a reduction of the magnitude of RAEs in female talents. A coach intervention might have decreased these RAEs.

• (RAEs) increase within higher selections in German handball.

## Background

In﻿﻿﻿ the last thirty years the phenomena of relative age effects (RAEs) have received considerable amount of attention in sport research [1–3]. For the most part, research has observed that relatively older athletes, those born at the beginning of their age group, are over-represented on youth (e.g., [4]) and then eventually elite adults levels of play [5, 6]. These effects exist in individual and a variety of team sports, and have been observed internationally (for reviews see [1, 2, 6]). The fact that the predominant age grouping system used in youth sport may create talent identification and selection biases, as well as potential barriers to participation, likely accounts for the continued interest in this topic.

In an attempt to better understand the factors that influence RAEs, Wattie et al. [3] proposed that it is necessary to consider the interaction of multiple factors. More specifically, they assert that the characteristics of the sport (e.g., rules and parameters of play), the individual (e.g., anthropometrics), and broader environmental factors all interact to influence specific RAEs. For example, it has been hypothesized that the cultural popularity (an environmental factor) of a sport may influence the number of youth participating, which then results in increased competition for available positions on a highly competitive teams [7]. The increased competition for available positions on a team, coupled with pressure to select teams that will succeed short term, may subsequently increase the selection pressures that favor relatively older athletes. While factors such as competition may influence RAEs, it is also important to consider how such factors interact with other relevant factors (i.e., sport, individual and environmental), such as sex and time.

Previous studies focusing on RAEs conducted in both male and female athletes revealed that sex can also be a moderator of RAEs [3]. Although RAEs are generally confirmed for both sexes over a range of sports, competition and developmental levels [1, 2], the magnitude of the effects appear different between male and female athletes [8]. Typically, the effect size in female athletes is found to be smaller when compared to those of male athletes [5], but female athletes population have also exhibited different types of RAE (e.g. an overrepresentation of the relatively younger gymnasts or the Q2-effect; [1]).

Several explanations for different RAEs between female and male athletes have been proposed. First, the higher participation number in male athletes is frequently suggested as a main reason for the larger RAEs observed in male athletes [8]. If a high number of athletes are competing for only a small number of positions within a team, this is likely to enhance the influence of relative age within a cohort [7–9]. Nevertheless, even when participation numbers are equal for female and male athletes in a certain context, sex differences in RAEs can appear [9]. Therefore, the number of participants alone does not clarify the variability between sexes completely. Second, the differences in growth and (pre-)maturation between girls and boys are proposed as explanations. These differences can cause a ‘time-shift’ in the importance of their influence on RAEs when comparing age-peer groups of both sexes. Smith et al. [1] showed indeed that in the female samples the highest risk for RAEs appeared in the younger age category (≤ 11 years) compared to the results of male samples (13–15 years). Additionally, the earlier appearance of RAEs in girls might cause a cascade of events, which can change the probability of the emergence and/or conservation of future relative age effects. Third, differences in the breadth of sports between sexes (e.g. media attention, sport-specific funding, cultural acceptance of athletes, level of physicality) is another proposed factor, which can interact with the timing of maturation. Entering puberty may be associated with negative outcomes in specifically female athletes, (e.g. increased body mass-to-height ratio) impacting performance in particular contexts, and other psycho-social concerns (e.g. body image). Vincent and Glamser [10] suggested that a stereotyped definition of femininity could make early maturing females less motivated to participate in sport, perhaps to conform to socially constructed gender roles [11]. Overall, as Wattie et al. (2015) suggested, it appears that relative age may be a more complex and variable constraint among female athletes than in male athletes. Consequently, it seems sensible to further explore the variability of RAEs between sexes in sports.

Additionally, surprisingly little attention has been given to the importance of replications in sports. For RAEs the closest study looking indirectly to replicate previous findings was conducted by Helsen and colleagues [12]. They found that after ten years RAEs were still the same in European football. One reason could be that “more than one replication study is needed for unambiguous tests of replication” [13]. In the case of RAE, many single replications in different sports and different ages exist, but the effect could sometimes be replicated and sometimes not replicated. Obviously, it depends on various factors like the chosen sport, the sex or the age of the athletes. However, to the best of our knowledge the stability of RAEs and its variation within in one sport and the same age has rarely been investigated. Wattie et al. [14] looked at RAEs in the NHL in 5-year increments from < 1900 to 1980. Data suggested that RAEs only emerged for players born from 1956 onward. Prior to that point there was no evidence of RAEs in the NHL. We propose that an examination of the stability of RAE in one sport requires the computation of an effect size and its confidence intervals of the effect size of a ‘baseline’ cohort. On conditions that the confidence interval does not include the null and RAEs are resilient within one age group, the effect size of every next cohort (e.g. in the subsequent years) has to be in the range of the original baseline cohort’s confidence interval.

## Aims of the Study

While previous work hoped for a reduction of relative age effects with education of the coaches, there were actually not many changes after a replication ten years later [12]. However, the stability and variability during these ten years was not tested before. Consequently, the first aim of this study was to test the stability and variability of within-year effects in handball players over time during national talent selection. A second aim was to check, if at the same context and time frame, the effects sizes are stronger in male compared to female talents. This could be expected from previous research in general [1], but also in handball specifically [8, 15, 16]. Thirdly, we wanted to investigate, if the next selection step for a junior national team, which occurs by the selections at these national talent camps, increases the within year effects compared to variability estimates from 16 years prior, as the competition hypothesis would suggest [8, 9, 17]. We assume that these selections increase the effects differently between the sexes, which previous studies have shown [16].

## Methods

### Sample

For this retrospective study, birth dates were provided for all participants of the talent selection camps by the German Handball Federation (Deutscher Handballbund e.V.) from 2008 to 2024. Additionally, we received the information which players were chosen during these talent selections for the youth national team. At these talent selection camps there is space for approximately 240 female and 240 male handball players per year. Approximately 10% of them are from a one-year younger group, which is why we have excluded them from our analyses. The boys were between 15 and 16 years of age, while the girls were from 14 to 15 years of age. Out of these 30–65 female and 30–65 male players were selected to the next talent selection camp, at which the players for the youth national teams were chosen. In the year 2021, the talent camp had to be canceled due to COVID restrictions. However, the selection had to occur for this year group and therefore the national coaches relied on the regional coaches to select their best players. These selections were discussed in online meetings and resulted in a first selection, that is reported here, but need to be considered under the special circumstances.

### Statistical Analyses

Given that the cut-off date in handball is the first of January, players’ birth months were re-coded to reflect his or her birth quartile (January to March = quartile 1, April to June = quartile 2, July to September = quartile 3 and October to December = quartile 4). Because previous research on birthdate distribution in Germany can be considered equal between quartiles, statistical analyses were conducted against this equal distribution [16]. Chi-square tests on goodness of fit were used to test for difference in birth distributions between the quartiles. This was done separately for sexes. All analyses were conducted with R 4.4.1. Additionally, we present the results for only the selected players to examine if this selection step increased or decreased within-year effects. The effect size *w* and פ were reported. According to Cohen [18] an effect size w of 0.10 represents a small effect, 0.30 a medium effect and 0.50 a large effect. Effects sizes of פ are small above 0.10, medium above 0.20, and large for above 0.30. Odds ratios between Q1 and Q4 are also reported to illustrate how many times more likely a player from Q1 is to be selected compared to Q4. For each effect size and odds ratios, 90% confidence interval was calculated based on the noncentral Chi-square distributions.^1^ To compare the OR_Q1/Q4_, the OR of all versus selected players and female versus male payers OR were transformed in r and Cohen’s q [18] was used to interpret the differences. According to Cohen [18] q < 0.10 represents no effect, 0.10 − 0.30 a small effect, 0.30 − 0.50 a medium effect and > 0.50 a large effect. For the description of stability and variability, the confidence intervals for the year 2008 were used for all participants, because sample sizes were large enough (female: *N* = 3,313; male: *N* = 3,460) to consider a valid range. For the selected players, who represent much smaller sample sizes (female: *N* = 723; male: *N* = 703), we used the mean confidence interval for overall years.

## Results

**Fig. 1:**
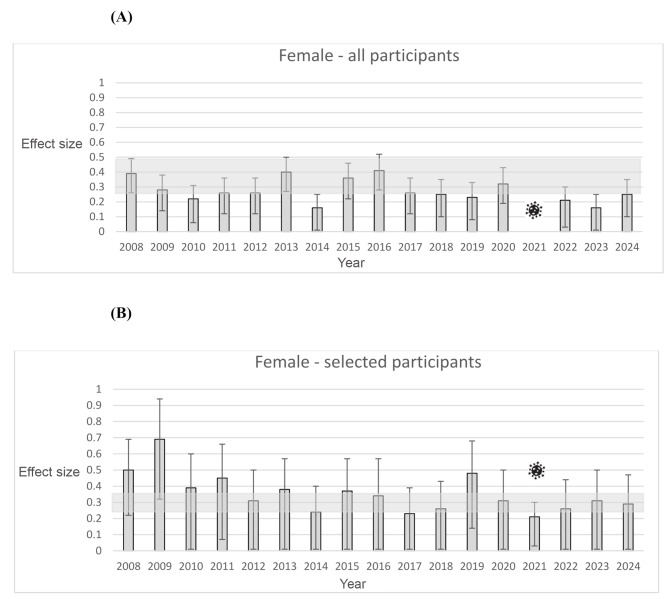
(**A**) Effect sizes for all female players. Error bars indicate 90% confidence intervals. The grey rectangle indicates the confidence interval borders for year 2008. The COVID symbol indicates, that in the year 2021 the talent camp had to be canceled due to COVID restrictions. (**B**) Effect sizes for selected female players. Error bars indicate 90% confidence intervals. The grey rectangle indicates the confidence interval borders overall years. The COVID symbol indicates, that in the year 2021 the talent camp had to be canceled due to COVID restrictions

The first aim of this study was to test for stability and variability of the within-year effects over seventeen years of time, and a second aim was to compare these trends between sexes. As can be seen in Table 1, significant RAEs were found for most of the female players (with exceptions in 2014, 2021, and 2023) and for all of the male players that participated at the talent selections in the year 2008 to 2024 (cf. Table 2). There was some variation in effect sizes for female and male talents. However, the comparison of the OR between all female and all male athletes, shows no effect respectively no significant difference, *q* = 0.09. As can be seen in Fig. 1a in light grey, for the female overall sample the effect sizes ranged from 0.16 (small) in year 2014 and 2023 to 0.41 (medium) for year 2016. This is mostly within the confidence intervals for the year 2008 (90% CI:0.26-0.49), which was the initially defined starting year for testing stability and variability.

**Table 1:** Birth quartile distributions (Q1-Q4) and Chi²-test results including effect sizes w and פ as well as odds ratios for Q1/Q4 and their confidence intervals differentiated by nomination and year of participation for female players

Nomination	Year	*n*	Q1 [%]	Q2 [%]	Q3 [%]	Q4 [%]	Χ²(3)	*p*	w	90% CI	פ	90% CI	OR Q1/Q4	90% CI
All participants	2008	215	39.07	25.12	24.19	11.63	32.46	< 0.001	0.39	[0.26, 0.49]	0.22	[0.15, 0.28]	4.87	[3.21, 7.41]
	2009	201	34.33	28.86	20.90	15.92	16.17	0.001	0.28	[0.14, 0.38]	0.16	[0.08, 0.22]	2.76	[1.85, 4.12]
	2010	208	33.65	25.48	19.71	21.15	9.81	0.020	0.22	[0.06, 0.31]	0.13	[0.04, 0.18]	1.89	[1.31, 2.73]
	2011	205	34.15	23.41	26.34	16.10	13.71	0.003	0.26	[0.12, 0.36]	0.15	[0.07, 0.21]	2.70	[1.82, 4.01]
	2012	209	32.06	28.23	25.36	14.35	14.52	0.002	0.26	[0.12, 0.36]	0.15	[0.07, 0.21]	2.82	[1.88, 4.22]
	2013	213	38.97	28.64	20.19	12.21	33.67	< 0.001	0.40	[0.27, 0.50]	0.23	[0.16, 0.29]	4.59	[3.03, 6.95]
	2014	211	31.28	25.59	21.80	21.33	5.36	0.147	0.16	[< 0.01, 0.25]	0.09	[< 0.01, 0.14]	1.68	[1.16, 2.43]
	2015	205	37.07	28.29	21.95	12.68	26.04	< 0.001	0.36	[0.22, 0.46]	0.21	[0.13, 0.27]	4.06	[2.67, 6.17]
	2016	204	40.20	27.45	20.10	12.25	34.55	< 0.001	0.41	[0.28, 0.52]	0.24	[0.16, 0.30]	4.81	[3.15, 7.34]
	2017	205	31.22	31.71	20.00	17.07	14.06	0.003	0.26	[0.12, 0.36]	0.15	[0.07, 0.21]	2.20	[1.49, 3.27]
	2018	202	33.17	28.71	18.32	19.80	12.30	0.006	0.25	[0.10, 0.35]	0.14	[0.06, 0.20]	2.01	[1.37, 2.94]
	2019	197	32.99	26.40	23.86	16.75	10.65	0.014	0.23	[0.08, 0.33]	0.13	[0.05, 0.19]	2.45	[1.64, 3.65]
	2020	203	36.95	27.59	19.70	15.76	21.33	< 0.001	0.32	[0.19, 0.43]	0.19	[0.11, 0.25]	3.13	[2.11, 4.66]
	2021	31	41.94	19.35	22.58	16.13	5.00	0.172	0.40	[< 0.01, 0.63]	0.23	[< 0.01, 0.36]	3.76	[1.38, 10.23]
	2022	197	32.99	21.32	25.89	19.80	8.30	0.040	0.21	[0.03, 0.30]	0.12	[0.02, 0.17]	1.99	[1.36, 2.93]
	2023	213	27.23	30.05	23.47	19.25	5.61	0.132	0.16	[< 0.01, 0.25]	0.09	[< 0.01, 0.15]	1.57	[1.07, 2.30]
	2024	194	31.44	23.20	29.90	15.46	12.39	0.006	0.25	[0.10, 0.35]	0.15	[0.06, 0.20]	2.51	[1.66, 3.79]
	Total	3313	34.26	26.83	22.58	16.33	225.46	< 0.001	0.26	[0.23, 0.29]	0.15	[0.13, 0.17]	2.67	[2.42, 2.95]
Selected participants	2008	56	39.29	30.36	25.00	5.36	13.86	0.003	0.50	[0.22, 0.69]	0.29	[0.13, 0.40]	11.43	[3.9, 33.49]
	2009	31	38.71	45.16	6.45	9.68	14.55	0.002	0.69	[0.32, 0.94]	0.40	[0.19, 0.54]	5.89	[1.83, 18.97]
	2010	41	39.02	29.27	17.07	14.63	6.32	0.097	0.39	[< 0.01, 0.60]	0.23	[< 0.01, 0.35]	3.73	[1.52, 9.16]
	2011	41	41.46	24.39	24.39	9.76	8.27	0.041	0.45	[0.07, 0.66]	0.26	[0.04, 0.38]	6.55	[2.38, 18.00]
	2012	43	30.23	18.60	34.88	16.28	4.16	0.244	0.31	[< 0.01, 0.50]	0.18	[< 0.01, 0.29]	2.23	[0.93, 5.33]
	2013	54	40.74	24.07	18.52	16.67	7.78	0.051	0.38	[< 0.01, 0.56]	0.22	[< 0.01, 0.32]	3.44	[1.62, 7.31]
	2014	52	30.77	25.00	28.85	15.38	2.92	0.404	0.24	[< 0.01, 0.40]	0.14	[< 0.01, 0.23]	2.44	[1.10, 5.45]
	2015	43	39.53	23.26	23.26	13.95	5.84	0.120	0.37	[< 0.01, 0.57]	0.21	[< 0.01, 0.33]	4.03	[1.66, 9.79]
	2016	27	29.63	33.33	25.93	11.11	3.07	0.380	0.34	[< 0.01, 0.57]	0.19	[< 0.01, 0.33]	3.37	[0.99, 11.44]
	2017	48	31.25	29.17	22.92	16.67	2.50	0.475	0.23	[< 0.01, 0.39]	0.13	[< 0.01, 0.23]	2.27	[1.00, 5.15]
	2018	58	29.31	32.76	22.41	15.52	4.07	0.254	0.26	[< 0.01, 0.43]	0.15	[< 0.01, 0.25]	2.26	[1.05, 4.84]
	2019	44	40.91	31.82	11.36	15.91	10.00	0.019	0.48	[0.14, 0.68]	0.28	[0.08, 0.40]	3.66	[1.57, 8.52]
	2020	47	36.17	27.66	21.28	14.89	4.66	0.198	0.31	[< 0.01, 0.50]	0.18	[< 0.01, 0.29]	3.24	[1.40, 7.49]
	2022	42	35.71	19.05	23.81	21.43	2.76	0.430	0.26	[< 0.01, 0.44]	0.15	[< 0.01, 0.25]	2.04	[0.90, 4.60]
	2023	47	31.91	23.40	31.91	12.77	4.66	0.198	0.31	[< 0.01, 0.50]	0.18	[< 0.01, 0.29]	3.20	[1.32, 7.76]
	2024	49	32.65	22.45	30.61	14.29	4.14	0.246	0.29	[< 0.01, 0.47]	0.17	[< 0.01, 0.27]	2.91	[1.26, 6.72]
	Total	723	35.41	27.11	23.37	14.11	67.69	< 0.001	0.31	[0.24, 0.36]	0.18	[0.14, 0.21]	3.34	[2.69, 4.15]

**Table 2:** Birth quartile distributions (Q1-Q4) and Chi²-test results including effect sizes w and פ as well as odds ratios for Q1/Q4 and their confidence intervals differentiated by nomination and year of participation for male players

Nomination	Year	*n*	Q1 [%]	Q2 [%]	Q3 [%]	Q4 [%]	Χ²(3)	*p*	w	90% CI	פ	90% CI	OR Q1/Q4	95% CI
All participants	2008	207	36.71	28.02	24.64	10.63	29.23	< 0.001	0.38	[0.25, 0.48]	0.22	[0.14, 0.28]	4.88	[3.14, 7.58]
	2009	216	34.26	25.93	26.85	12.96	20.30	< 0.001	0.31	[0.17, 0.41]	0.18	[0.10, 0.23]	3.50	[2.33, 5.26]
	2010	218	42.66	27.06	18.35	11.93	46.33	< 0.001	0.46	[0.34, 0.56]	0.27	[0.20, 0.33]	5.49	[3.64, 8.29]
	2011	213	36.15	28.17	20.19	15.49	21.12	< 0.001	0.31	[0.18, 0.41]	0.18	[0.11, 0.24]	3.09	[2.09, 4.56]
	2012	211	39.81	23.22	24.17	12.80	31.41	< 0.001	0.39	[0.26, 0.49]	0.22	[0.15, 0.28]	4.51	[2.99, 6.79]
	2013	217	35.02	31.34	22.12	11.52	28.70	< 0.001	0.36	[0.24, 0.46]	0.21	[0.14, 0.27]	4.14	[2.72, 6.31]
	2014	213	37.56	30.99	21.13	10.33	36.11	< 0.001	0.41	[0.29, 0.51]	0.24	[0.16, 0.30]	5.22	[3.37, 8.09]
	2015	217	30.88	26.73	26.27	16.13	10.23	0.017	0.22	[0.07, 0.31]	0.13	[0.04, 0.18]	2.32	[1.58, 3.42]
	2016	212	38.21	29.25	17.92	14.62	29.70	< 0.001	0.37	[0.25, 0.48]	0.22	[0.14, 0.27]	3.61	[2.43, 5.36]
	2017	209	35.89	28.23	22.49	13.40	22.56	< 0.001	0.33	[0.20, 0.43]	0.19	[0.11, 0.25]	3.62	[2.40, 5.45]
	2018	206	43.69	26.70	12.62	16.99	46.93	< 0.001	0.48	[0.35, 0.58]	0.28	[0.20, 0.34]	3.79	[2.59, 5.56]
	2019	217	45.16	23.04	19.82	11.98	52.66	< 0.001	0.49	[0.37, 0.60]	0.28	[0.21, 0.34]	6.05	[4.01, 9.12]
	2020	217	33.18	26.27	22.58	17.97	10.74	0.013	0.22	[0.08, 0.32]	0.13	[0.04, 0.18]	2.27	[1.56, 3.30]
	2021	35	54.29	25.71	14.29	5.71	18.83	< 0.001	0.73	[0.40, 0.98]	0.42	[0.23, 0.56]	19.59	[5.23, 73.45]
	2022	198	39.90	25.25	19.19	15.66	21.17	< 0.001	0.33	[0.19, 0.43]	0.21	[0.14, 0.27]	3.58	[2.40, 5.34]
	2023	218	34.86	29.82	18.81	16.51	20.13	< 0.001	0.30	[0.17, 0.40]	0.18	[0.10, 0.23]	2.71	[1.85, 3.96]
	2024	236	35.59	25.42	27.54	11.44	28.58	< 0.001	0.35	[0.23, 0.44]	0.20	[0.13, 0.26]	4.28	[2.86, 6.41]
	Total	3460	37.60	27.20	21.53	13.67	420.74	< 0.001	0.35	[0.32, 0.38]	0.20	[0.18, 0.22]	3.81	[3.44, 4.20]
Selected participants	2008	35	37.14	34.29	20.00	8.57	7.40	0.060	0.46	[< 0.01, 0.69]	0.27	[< 0.01, 0.40]	6.30	[2.00, 19.86]
	2009	44	52.27	18.18	22.73	6.82	19.82	< 0.001	0.67	[0.38, 0.89]	0.39	[0.22, 0.51]	14.97	[4.97, 45.06]
	2010	58	48.28	25.86	15.52	10.34	19.66	< 0.001	0.58	[0.33, 0.77]	0.34	[0.19, 0.45]	8.09	[3.53, 18.56]
	2011	43	34.88	25.58	20.93	18.60	2.67	0.445	0.25	[< 0.01, 0.43]	0.14	[< 0.01, 0.25]	2.34	[1.02, 5.39]
	2012	55	45.45	20.00	23.64	10.91	14.16	0.003	0.51	[0.23, 0.70]	0.29	[0.13, 0.40]	6.81	[2.94, 15.75]
	2013	44	40.91	31.82	18.18	9.09	10.55	0.014	0.49	[0.16, 0.70]	0.28	[0.09, 0.40]	6.92	[2.55, 18.80]
	2014	35	37.14	34.29	14.29	14.29	6.49	0.090	0.43	[< 0.01, 0.65]	0.25	[< 0.01, 0.38]	3.55	[1.33, 9.46]
	2015	31	45.16	16.13	19.35	19.35	6.81	0.078	0.47	[< 0.01, 0.71]	0.27	[< 0.01, 0.41]	3.43	[1.32, 8.91]
	2016	38	50.00	23.68	13.16	13.16	13.79	0.003	0.6	[0.27, 0.83]	0.35	[0.16, 0.48]	6.60	[2.55, 17.11]
	2017	48	31.25	35.42	25.00	8.33	8.17	0.043	0.41	[0.05, 0.61]	0.24	[0.03, 0.35]	5.00	[1.84, 13.59]
	2018	44	56.82	25.00	6.82	11.36	26.91	< 0.001	0.78	[0.50, 1.00]	0.45	[0.29, 0.58]	10.26	[4.06, 25.96]
	2019	50	56.00	24.00	10.00	10.00	28.24	< 0.001	0.75	[0.49, 0.96]	0.43	[0.28, 0.55]	11.45	[4.63, 28.34]
	2020	44	31.82	22.73	27.27	18.18	1.82	0.611	0.2	[< 0.01, 0.36]	0.12	[< 0.01, 0.21]	2.10	[0.91, 4.84]
	2022	41	31.71	29.27	31.71	7.32	6.90	0.075	0.41	[< 0.01, 0.62]	0.24	[< 0.01, 0.36]	5.88	[1.90, 18.21]
	2023	47	38.30	29.79	14.89	17.02	6.87	0.076	0.38	[< 0.01, 0.58]	0.22	[< 0.01, 0.33]	3.03	[1.35, 6.78]
	2024	46	32.61	26.09	34.78	6.52	9.13	0.028	0.45	[0.11, 0.65]	0.26	[0.06, 0.37]	6.94	[2.29, 21.05]
	Total	703	42.11	26.32	19.91	11.66	140.04	< 0.001	0.45	[0.38, 0.51]	0.26	[0.22, 0.29]	5.51	[4.37, 6.94]

The third aim of this study was to explore the effect of the next selection step in male and female athletes relative to the original baseline cohort’s variability of effects (i.e., in 2008), and to compare male and female effects. For the selected female players this range of effect sizes was even bigger, between 0.23 in year 2017 to 0.69 in the year 2008 (medium grey in Fig. 1b). This is not within the limits of the confidence intervals for the overall years (90% CI: 0.23-0.29). For the overall sample, there seems to be no trend for effect sizes, while for the selected players a reduction of effect sizes over the years is visible. Comparing the OR of all players to the selected players also reveal no difference, *q* = 0.06.

**Fig. 2:**
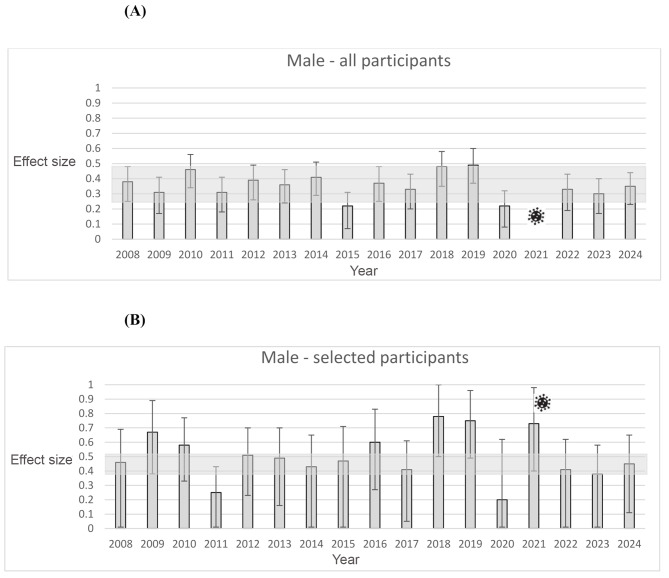
(**A**) Effect sizes for all male players. Error bars indicate 90% confidence intervals. The grey rectangle indicates the confidence interval borders year 2008. The COVID symbol indicates, that in the year 2021 the talent camp had to be canceled due to COVID restrictions. (**B**) Effect sizes for selected male players. Error bars indicate 90% confidence intervals. The grey rectangle indicates the confidence interval borders overall years. The COVID symbol indicates, that in the year 2021 the talent camp had to be canceled due to COVID restrictions

For the male athletes, in Fig. 2a the light grey bars indicate a range of effect sizes for all players between 0.22 in years 2015 and 2020 to 0.49 in year 2019. Here the effect size of the year 2019 is slightly above and the years 2015 and 2020 slightly below the limits of the confidence interval from the year 2008 (90% CI: 0.25-0.48]. Additionally, the range only differs slightly in contrast to the female talents and both samples have small to medium effect sizes here. No trend seems observable for all male players.

The range of effect sizes for the selected male players is between 0.20 in the year 2020 to 0.78 in the year 2018. Most effect sizes of the different years are outside the limits of the confidence interval overall years (90% CI: 0.38- 0.51]. As for the female talents, this range is bigger than within the overall sample. Overall, there seems to be no trend in this sample as well. Comparing the OR of all male players to the selected male players revealed no significant difference, *q* = 0.09. However, comparing the difference of the effects for the female and the male selected players revealed a small effect, q = 0.13.

## Discussion

This study had three aims. The first aim was to test the stability and variability of within-year effects in young handball talents [8, 15, 17]. As can be seen in Fig. 2A and B, for the male athletes most of the effects sizes between 2019 and 2024 were within the confidence intervals for the effect size from 2008. For all participants there is a rather small variation in these effect sizes and they are all in the area of a small to medium effect size. For the selected male athletes, the effect sizes are in general higher than for the overall sample, which is in line with the competition hypothesis [3, 7, 9, 17], however no clear developmental trend can be recognized. For all female talents, again, most of the small to medium effects sizes between 2009 and 2024 were within the confidence intervals for the effect size from 2008. The most notable finding can be seen in Fig. 1b. Here a clear change of the effect sizes for the selected female athletes can be seen. While in the beginning of the observed time period the effect sizes are larger, they tend to become smaller with singular exceptions. One possible explanation might be the hypothesis Helsen and colleagues presented [12]. The education of the coaches might have helped to decrease the relative age effects especially on the national selection level. Previous research by Schorer and colleagues had shown consistent RAEs in this selection level and age group in German handball [8, 15, 16]. Due to the close collaboration of two of the authors with the German handball federation, these results were presented at a sport-specific workshops with the national coaches in attendance with follow-up discussions with the new national coaches [19]. While this topic might have been taken seriously by the coaches for the female athletes, it may have been neglected by the coaches for the male players.

The second aim of this study was to compare the effect sizes for male and female athletes. Previous research had shown in general higher effect sizes for male athletes than for females [1, 2]. The same trend holds true for handball [8, 15, 16]. This trend remains with a mean higher effect size for the males than for the female players, although the difference is rather small. Larger effects size differences were observed between male and female selected players. Especially since the previously described change for the female athletes with time, the female players show only small to medium effect sizes, while for the male the mean effect size is large. As previous studies have discussed, this might be best explained by the higher competition for the male spots than for the female spots, because the number of children playing handball as boys is substantially larger than girls [8, 15].

The third aim of the study was to test, if the next selection step for a youth national team, which occurs by the selections at these national talent camps, increased the within year effects as the competition hypothesis would suggest [8, 9, 17]. Previous studies have shown that this was the case previously [16]. As can be seen in the comparisons of the A- and B-figures this trend was replicated for the male handball players. The same holds for the female athletes in the beginning of the analyzed time period. The reason why this is not the case anymore afterwards, can only be speculated, as we have done above.

Taken together, this study provides an interesting picture of the variability and stability of relative age effects over seventeen years. Ultimately, these findings reinforce the importance of considering novel context-specific interactions between multiple developmental factors [3], such as absolute age, relative age, sex, specific sport structures, and broader athlete development milestones. While for the male athletes, trends remain rather stable, for the female talents a shift could be observed that needs further investigations. This might be a first clue for a possible solution, that previously had been dismissed. The question arises, why this was possible here for the female athletes, but not for the males here or the soccer players in Europe [12]? Previous research by Mann et al. [20] showed that age-ordered shirt numbering was able to reduce the effect. Further experimental research will be needed to understand these processes better. Additionally, future research might have to take a longitudinal look at the development of these trends for the same athletes during their childhood as advocated by Schorer et al. [21]. This might provide a clearer insight into these developmental processes and RAE mechanisms.

## Data Availability

The data can be requested via email from the first author on reasonable request.

## Footnotes

1 The non-centrality parameter for the non-central distributions was calculated under which a particular chi²-value cuts off an area of α% (here: 5%) to the right (λ_low_) and to the left (λ_low_). Based on these calculations, the lower (*w*_low_) and upper (*w*_upp_) bound of the 90% confidence interval on Cohen’s w are obtained via the formula *w*_low_ = sqrt[λ_low_/N] and *w*_upp_ = sqrt[λ_upp_/N], respectively.

## Abbreviations

RAE: Relative age effect
NHL: North-American Hockey League
